# Lymph Node Ratio Predicts Recurrence in Patients with Papillary Thyroid Carcinoma with Low Lymph Node Yield

**DOI:** 10.3390/cancers15112947

**Published:** 2023-05-27

**Authors:** Il Ku Kang, Joonseon Park, Ja Seong Bae, Jeong Soo Kim, Kwangsoon Kim

**Affiliations:** 1 Department of Surgery, Kyung Hee University Hospital at Gangdong, College of Medicine, Kyung Hee University, Seoul 05278, Republic of Korea; kik5304@gmail.com; 2 Department of Surgery, College of Medicine, The Catholic University of Korea, Seoul 06591, Republic of Korea; joonsunny@naver.com (J.P.); drbae@catholic.ac.kr (J.S.B.); btskim@catholic.ac.kr (J.S.K.)

**Keywords:** papillary thyroid carcinoma, lymph node ratio, lymph node yield, recurrence

## Abstract

**Simple Summary:**

In a retrospective study, researchers analyzed data from 909 patients with papillary thyroid carcinoma (PTC) who underwent thyroidectomy and neck dissection. The aim was to validate the relationship between lymph node ratio (LNR) and recurrence in patients with fewer than six lymph nodes examined after surgery. Over an average follow-up of 127.24 ± 33.6 months, recurrences occurred in 5.1% of the patients. The analysis identified an LNR ≥ 0.29 as an independent prognostic factor for recurrence. This means that patients with an LNR equal to or higher than 0.29 had a higher likelihood of recurrence compared to those with a lower LNR. These findings highlight the importance of considering LNR as a valuable prognostic marker for PTC patients with limited lymph node examination. Incorporating LNR into risk assessment can improve recurrence prediction and help guide treatment strategies. However, further prospective studies are needed to validate these results and determine their clinical implications in managing PTC patients.

**Abstract:**

The American Thyroid Association risk stratification system suggests that having >5 metastatic lymph nodes (LNs) increase the recurrence risk in patients with papillary thyroid carcinoma (PTC). However, little is known about PTC with <5 harvested LNs. This study aimed to stratify patients with low-LN-yield (low-LNY) PTC based on lymph node ratios (LNRs). From 2007 to 2017, 6317 patients who underwent thyroidectomies were diagnosed with PTC at Seoul St. Mary’s Hospital, and 909 patients with low LNYs were included in the study. Tumor recurrence was compared based on LNR. The LNR cutoff was determined using a receiver operating characteristic curve. Forty-six patients (5.1%) experienced recurrences over a mean follow-up period of 127.24 ± 33.6 months (range, 5–190 months). The cutoff for the low-LNR (*n* = 675) and high-LNR (*n* = 234) groups was 0.29 (AUC = 0.676, 95% CI = 0.591–0.761, *p* < 0.001). The recurrence rate was significantly higher in the high-LNR group compared to the rate in the low-LNR group (12.4% vs. 2.5%, *p* < 0.001). Multivariate analysis using Cox regression revealed that tumor size and LNR ≥ 0.29 were independent prognostic factors for recurrence. Therefore, LNR can be utilized to stratify the risk of recurrence in patients with low-LNY PTC.

## 1. Introduction

Thyroid cancer rates in the United States rose until the mid-2010s and then declined [1]. A 2020 study on the topic revealed that the incidence rates of thyroid cancer were 10.1 per 100,000 women and 3.1 per 100,000 men, while the mortality rates were 0.5 per 100,000 women and 0.3 per 100,000 men [2]. The most common type of thyroid cancer, papillary thyroid carcinoma (PTC), makes up 80–90% of cases and generally has a favorable prognosis [3]. In a retrospective study of 5897 patients with PTC, Ito et al. demonstrated that cancer-specific mortality was only 2% for a median follow-up of 177 months [4]. Although PTC has favorable survival rates, the recurrence rate is 8–23% after surgery, and lymph node (LN) metastasis is one of the risk factors affecting recurrence [5].

The number of LNs surgically retrieved is referred to as the lymph node yield (LNY). Three factors affect LNY [6]. Surgeons have different strategies for the extent of nodal dissection based on oncological outcomes and possible complications. Pathologists microscopically find and count LNs in person. Finally, patients have different anatomy in terms of the number of LNs.

According to the American Thyroid Association (ATA) surgical affairs committee task force 2015 guidelines, ≤5 pN1 micrometastases of <0.2 cm in the largest dimension are classified as low-risk N1 disease with a recurrence rate of <5% [7,8]. In contrast, the original risk stratification system in the ATA 2009 guidelines classified any loco-regional metastases as intermediate risk [9]. Although this modification decreased the number of patients upstaged and over-treated, risk is difficult to determine in patients with low LNY based on the ATA stratification system. For example, in patients with only two LNs examined, one metastatic node in the pathologic report does not mean ATA low risk. A limited number of LN examinations may lead to occult LN metastasis despite the diagnosis of pathologic N0. Robinson et al. analyzed 78,724 patients with PTC with ≥ 1 LNs examined after surgery and revealed that six, nine, and eighteen nodes would need to be examined for patients with T1b, T2, and T3 disease to rule out occult nodal disease with 90% confidence [10].

The lymph node ratio (LNR) is defined as the number of metastatic LNs divided by the number of LNs collected. A systematic review of nine retrospective studies showed that LNR was an independent predictor of loco-regional recurrence in metastatic PTC [11]. Furthermore, Parvathareddy et al. reported that LNR was a better predictor of tumor recurrence than the American Joint Committee on Cancer (AJCC) N stage (odds ratio 1.96 vs. 1.30, *p* value 0.0184 vs. 0.3831) [12]. The aim of this study was to validate the relationship between LNR and the risk of recurrence in patients with PTC and only one to five LNs examined.

## 2. Materials and Methods

### 2.1. Patients

A total of 6317 patients who underwent thyroidectomies at Seoul St. Mary’s Hospital were diagnosed with PTC between January 2007 and December 2017. Among them, 909 patients with low LNYs were selected for inclusion in the study. The study included patients who underwent total thyroidectomy and central neck lymph node dissection (CLND), as our institution performed prophylactic CLND for all PTC patients during the study period. Patients with lateral neck metastasis or distant metastasis were not included in the study. The Institutional Review Board of Seoul St. Mary’s Hospital, The Catholic University of Korea, approved the study protocol (IRB No: KC23RISI0055). Informed consent was not required for this retrospective study and was waived by the committee.

### 2.2. Postoperative Management and Follow-Up

All patients included in the study were monitored in accordance with the management guidelines provided by ATA [7,9]. Initially, follow-up visits occurred every 3 to 6 months, and later extended to once a year for patients who showed no signs of recurrence. The follow-up process involved a physical examination, neck ultrasonography, thyroid function test, serum thyroglobulin (Tg) test, and anti-Tg antibody test. Radioactive iodine (RAI) ablation was conducted 10–12 weeks after total thyroidectomy, and whole-body scans were performed 5–7 days after RAI ablation for patients with intermediate- and high-risk differentiated thyroid cancer. The frequency of follow-up visits and imaging tests varied based on the individual’s clinical progress.

When recurrent disease was suspected on routine follow-up examinations, additional diagnostic imaging studies, including neck computed tomography, were performed to determine the location and extent of the suspected recurrence. Recurrence was suspected when positive findings were noted on imaging studies or serum Tg levels increased significantly (≥50%). Recurrence was confirmed using ultrasound-guided fine-needle aspiration cytologies or surgical biopsies.

### 2.3. Statistical Analyses

Mean and standard deviation were used to present continuous variables, while numbers and percentages were used for categorical variables. Student’s *t*-tests were employed to compare continuous variables between the high- and low-LNR groups. Chi-squared or Fisher’s exact tests were used for the comparison of categorical variables. Univariate and multivariate Cox regression analyses were conducted to identify risk factors associated with recurrence. Receiver operating characteristic (ROC) curve analysis was employed to determine the optimal cutoff value for LNR in predicting recurrence, and sensitivity, specificity, and the area under the curve (AUC) with a 95% confidence interval (CI) were calculated. Statistical analyses were performed using IBM SPSS Statistics (version 24.0, IBM Corp., Armonk, NY, USA). Two-sided *p* values less than 0.05 were considered statistically significant.

## 3. Results

### 3.1. Baseline Clinicopathological Characteristics

The baseline clinicopathological characteristics of the patients are shown in Table 1. The mean follow-up duration for the 909 patients was 127.24 ± 33.6 months (range: 5–190 months), during which 46 patients (5.1%) experienced recurrence after their initial surgery. The mean patient age was 49.4 ± 12.6 years, and patients ≥55 years accounted for 35.5% of the patients. Females accounted for 80.6% of the patients. The mean tumor size was 0.92 ± 0.6 cm. Tumors were multifocal in 41.0% of patients. Microscopic and gross extrathyroidal extensions (ETE) were observed in 41.3% and 5.1% of patients, respectively. Of the 777 patients who were tested for BRAF V600E mutations, 659 patients (84.8%) were positive. The mean number of metastatic positive LNs was 0.6 ± 1.0 out of 3.3 ± 1.4 (range, 1–5) LNs examined. Postoperative RAI ablation was performed in 547 (60.2%) patients.

### 3.2. Comparison of Clinicopathological Characteristics according to LNR

**Figure 1:**
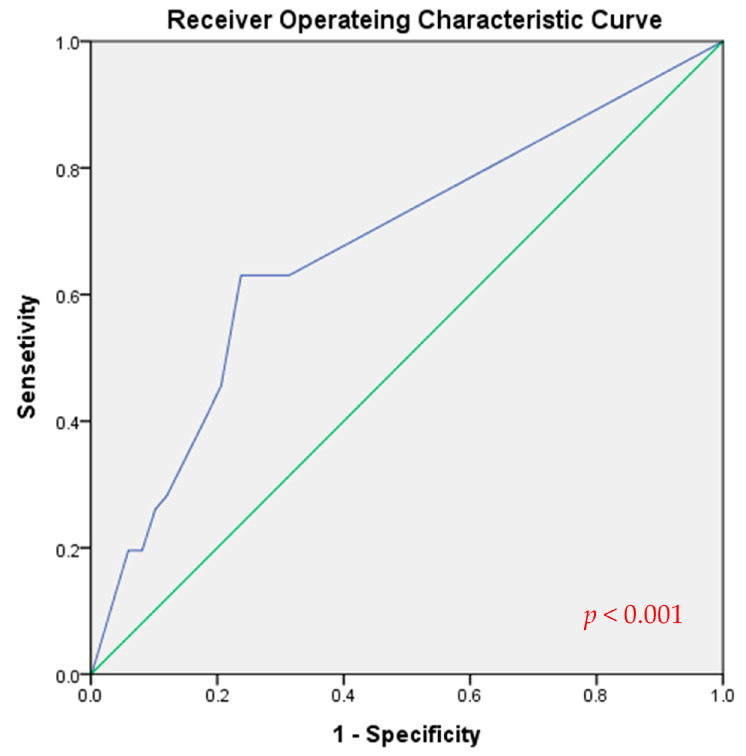
Receiver operating characteristic curve for lymph node ratio predicting recurrence.

Table 2 presents the clinicopathological characteristics of patients in the high-LNR (*n* = 234) and low-LNR group (*n* = 675). The cutoff value for LNR, determined by ROC curve analysis, was 0.29 (AUC = 0.676, 95% CI = 0.591–0.761, *p* < 0.001) as shown in Figure 1. The high-LNR group had a significantly younger mean age compared to the low-LNR group (47.17 ± 13.2 vs. 50.21 ± 12.3 years, *p* = 0.001), while the proportion of female patients was similar between the two groups (77.8% vs. 81.6%, *p* = 0.212). Tumor sizes were significantly larger in the high-LNR group compared to the low-LNR group (1.04 ± 0.6 vs. 0.87 ± 0.6, *p* < 0.001). The high-LNR group had a higher percentage of patients with lymphatic invasion (108/217, 49.8% vs. 55/559, 9.0%, *p* < 0.001) compared to the low-LNR group. No significant differences were observed in terms of multifocality, ETE, vascular and perineural invasion, and BRAF mutations between the two groups. The high-LNR group had a significantly higher mean number of positive LNs compared to the low-LNR group (2.0 ± 1.1 vs. 0.1 ± 0.3, *p* < 0.001), while the number of examined LNs was similar between the two groups (3.2 ± 1.3 vs. 3.3 ± 1.4, *p* = 0.485). The rate of postoperative RAI ablation therapy was significantly higher in the high-LNR group compared to the low-LNR group (91.5% vs. 49.3%, *p* < 0.001). According to the eighth edition of the staging system by the American Joint Committee on Cancer/Union for International Cancer Control, the high-LNR group had a significantly higher TNM stage compared to the low-LNR group (stage II, 26.5% vs. 5.8%, *p* < 0.001), while the T stage rate showed no significant difference (*p* = 0.129). About one out of ten patients in the low-LNR group developed LN metastasis (N1a). The recurrence rate was significantly higher in the high-LNR group compared to the low-LNR group (29/234, 12.4% vs. 17/675, 2.5%, *p* < 0.001).

### 3.3. Univariate and Multivariate Analyses of the Risk Factors for Recurrence

**Figure 2:**
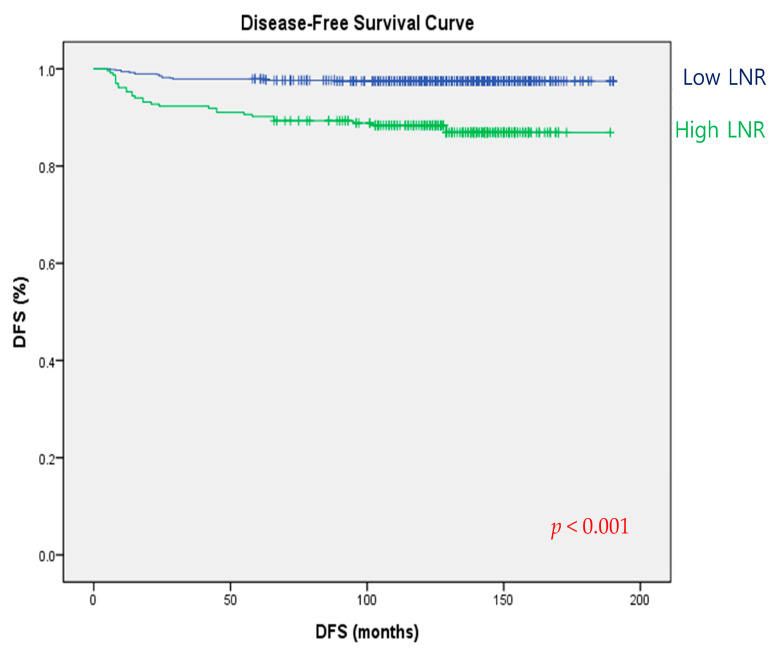
Disease-free survival curves according to lymph node ratio (log-rank *p* < 0.001).

The clinicopathological parameters that were significantly associated with recurrence are listed in Table 3. Univariate analysis revealed that tumor size (HR = 2.026, 95% CI 1.621–2.533, *p* < 0.001), positive LNs (1.505, 1.251–1.811, *p* < 0.001), LNR (2.832, 1.837–4.365, *p* < 0.001), minimal and gross ETE vs. no ETE (2.224, 1.173–4.219, *p* = 0.014; 4.613, 1.789–11.894, *p* = 0.002), lymphatic invasion (3.469, 1.861–6.468, *p* < 0.001), LNR ≥ 0.29 (5.173, 2.843–9.414, *p* < 0.001), T2 and T3b vs. T1 (4.539, 1.904–10.820, *p* = 0.001; 3.417, 1.434–8.142, *p* = 0.006), N1a (3.470, 1.907–6.315, *p* < 0.001), and stage II (2.335, 1.158–4.706, *p* = 0.018) were significantly associated with recurrence. Postoperative RAI was also a predictor for recurrence (49.684, 5.076–486.260, *p* = 0.001). Multivariate analysis revealed that tumor size and LNR > 0.29 were independent prognostic factors predicting recurrence (2.111, 1.596–2.792, *p* < 0.001; 6.191, 3.176–12.066, *p* < 0.001). The Kaplan–Meier analysis demonstrated a significant difference in DFS between the high-LNR and low-LNR groups (log-rank *p* = 0.001) (Figure 2).

### 3.4. Subgroup Analysis for Patients with Postoperative RAI Ablation

Table 4 shows the comparisons of clinicopathological factors according to LNR for patients who underwent postoperative RAI ablation (*n* = 547). A cutoff of LNR 0.29 predicted recurrence (high-LNR, *n* = 214; low-LNR, *n* = 333). Patients with high LNRs were more likely to be younger than patients with low LNRs (47.17 ± 13.2 vs. 50.26 ± 11.6, *p* = 0.001). Lymphatic invasion occurred significantly more in the high-LNR group compared with the low-LNR group (50.8% vs. 15.8%, *p* < 0.001). The mean number of positive LNs for metastasis was significantly higher in the high-LNR group compared with the low-LNR group (2.0 ± 1.1 vs. 0.2 ± 0.4, *p* < 0.001). All the patients in the high-LNR group were stage N1a, but only 60 out of 333 patients in the low-LNR group were stage N1a (100% vs. 18.0%, *p* < 0.001). The stage II rate was also significantly higher in the high-LNR group compared with the low-LNR group (24.8% vs. 8.7%, *p* < 0.001). Twenty-nine and seventeen patients had tumor recurrence in the high- and low-LNR groups, respectively (13.6% vs. 5.1%, *p* = 0.001).

## 4. Discussion

This study demonstrated that LNR can be used to stratify the risk of recurrence in patients with PTC with an LNY of five or fewer. LNR ≥ 0.29 was an independent prognostic factor for the risk of recurrence in patients with low-LNY PTC (HR 6.191, 95% CI 3.176–12.066, *p* < 0.001). The recurrence rate in the high-LNR group was significantly higher than the recurrence rate in the low-LNR group (12.4% vs. 2.5%, *p* < 0.001). In the subgroup analysis of patients who underwent postoperative RAI ablation, the high-LNR group developed more recurrence than the low-LNR group (13.6% vs. 5.1%, *p* = 0.001). Our results may help risk-stratify patients with PTC with a small number of harvested LNs, which was difficult under the current ATA risk stratification system.

Cervical LN metastasis significantly affects the likelihood of local recurrence and survival outcomes in patients with PTC [13]. Thus, a great deal of research has been conducted to investigate the various risk factors associated with LN metastasis in PTC. Several risk factors significantly associated with LN metastasis in patients with PTC were identified in previous studies, including male gender, younger age, larger tumor size, multifocality, ETE, and lymphovascular invasion [14,15,16,17,18]. The identification of these risk factors facilitates the development of effective screening, diagnosis, and treatment strategies for patients with PTC at risk of LN metastasis. Furthermore, understanding the underlying mechanisms behind these risk factors provides valuable insights into the pathogenesis of PTC and facilitates the identification of novel therapeutic targets for the disease. Despite the progress made in identifying risk factors associated with LN metastasis in PTC, more research is still needed to fully elucidate the complex interplay between these parameters and their impact on patient outcomes. Further investigation will undoubtedly improve our understanding of PTC and improve patient care and outcomes.

Five LNs or less was the criterion set for a small number of lymph nodes examined, and only patients with PTC and one to five LNs retrieved after total thyroidectomy with bilateral central LN dissection were included in the study. The ATA risk stratification did not indicate an adequate number of LNs examined to apply the more-than-five-metastases criterion in the system [7]. Regarding PTC with between one and five pathologic LNs examined, we have two conflicting perspectives; The PTC may not be aggressive because only one to five metastatic LNs were identified, while PTC with a low LNY is related to a lesser extent of surgical resection, which means a higher rate of recurrence. We recently performed retrospective studies regarding LNR in PTC and recurrent thyroid cancer [19,20], and we wondered if LNR is associated with the prognosis of patients with PTC when a small number of LNs are examined.

In our study, 46 patients out of 909 (5.1%) patients experienced tumor recurrence during a follow-up of 127.24 ± 33.6 months. A meta-analysis that included six studies with 2939 patients surveyed between 2022 and 2013 (mean follow-up, 10.9 ± 3.4 years) demonstrated a recurrence rate of 4.4% in the total-thyroidectomy group (2134 patients) [21], which is not significantly different from our finding.

We performed a subgroup analysis to determine if postoperative RAI therapy affected differences in the recurrence rates between the groups. Postoperative RAI ablation was once one of the main therapeutic strategies in patients with thyroid cancer but does not impact the recurrence rate, particularly in patients with low-risk thyroid cancer [21,22,23]. In our subgroup analysis of patients who underwent postoperative RAI, we observed a difference in recurrence between the high-LNR and low-LNR groups.

Several groups reported data on the effects of LNY on prognosis in patients with PTC [6,24,25,26,27]. Yu et al. showed that higher LNY in CLND was associated with lower recurrence rates in PTC [6]. Heaton et al. reported that higher LNY in central neck dissections (2.5 vs. 10.3; *p* < 0.0001) and lateral neck dissections (10.5 vs. 24.6; *p* < 0.0001) were associated with lower rates of recurrence in patients with PTC [24]. Noel et al. showed that the mean LNY of patients with central and lateral neck persistence was significantly lower than the mean LNY in patients with disease-free survival (4.8 vs. 11.9; OR = 0.69, 95% CI 0.59–0.8; *p* < 0.001) [25]. However, some studies showed different results. Beal et al. demonstrated that increasing LNY was associated with worse survival according to a multivariate analysis [26]. Vas Nunes et al. identified no prognostic implications for LNY [27]. Since each surgical group has different methods and strategies regarding the extent of central neck dissections, different findings between studies can occur.

Several studies focused on the relationship between LNR and the prognosis of patients with PTC, including the number and extent of metastatic LNs. Jeon et al. found that LNR > 0.4 was an independent risk factor for recurrence in pathological N1a PTC [28]. Schneider et al. revealed that LNR ≥ 0.42 was associated with disease-specific mortality (HR = 4.33; 95% CI 1.68–11.18; *p* < 0.01) [29]. Pyo et al. reported that LNR ≥ 0.44 significantly correlated with worse 5- and 10-year disease-free survival rates [30]. Several studies also suggested a lower cutoff for LNR. Vas Nunes et al. showed that LNR was an independent prognostic factor in PTC and suggested a cutoff of 0.3 [27]. Zheng et al. found that LNR ≥ 0.31 was significantly associated with recurrence in a multivariate analysis (HR = 11.23) [31]. Weitzman et al. reported that a cutoff of LNR 0.3 predicted the risk of recurrence in PTC [32]. Although these findings were based on retrospective studies that have biases, they are in line with the results of this study, showing that LNR ≥ 0.29 was an independent prognostic factor in PTC with less-than-six LNs retrieved.

There were several limitations due to the retrospective analysis. We included patients who underwent total thyroidectomy and RAI ablation therapy from 2007 to 2017. The ATA management guidelines were released in 2015; thus, the majority of patients were treated according to the former guidelines from 2009 [9], except for patients who underwent surgery between 2016 and 2017. Therefore, thyroid lobectomies were performed and followed up instead of complete thyroidectomies unless the patients had >5 pathologic LN metastases or metastases >2 mm in the largest diameter, after the 2015 guidelines [7]. In addition, of the patients included in this study, 5.1% (46 patients) of patients had gross ETE and were classified as high-risk according to the ATA guidelines. We may have clarified the study subjects to stratify low-risk or intermediate-risk patients if we ruled out patients with gross ETE. We focused on the risk stratification system in the latest ATA guidelines; thus, we may have missed some parameters related to recurrences, such as the diameter of the largest focus of LNs, aggressive histology, and TERT (telomerase reverse transcriptase) promoter mutations. Finally, our data are difficult to apply to generalized thyroid cancer patients because over 90% fall into T1 and the mean tumor size is below 1 cm.

Nonetheless, our study has several strengths. We analyzed 909 patients and followed patients for 127.24 ± 33.6 months. To our best knowledge, our study is the first to verify the association between LNR and recurrence with long-term data, including patients with PTC who underwent surgery. The recent trend in PTC treatment is not to perform total thyroidectomy. Thus, future research should address the patients treated with thyroid lobectomies and unilateral central LN dissections.

## 5. Conclusions

LNR ≥ 0.29 was an independent predictor of recurrence in patients with PTC with LNY ≤ 5. This finding may help follow-up patients with PTC after total thyroidectomy and cervical LN dissection. Further prospective studies are needed to substantiate our results.

## Figures and Tables

**Table 1:** Baseline characteristics of the study patients.

Total 909 Patients	
Age (years)	49.43 ± 12.6 (range, 20–83)
≥55	323 (35.5%)
<55	586 (64.5%)
Male: Female	1: 4.16
Male	176 (19.4%)
Female	733 (80.6%)
Tumor size (cm)	0.92 ± 0.6 (range, 0.08–5.5)
Multifocal (uni/bilateral)	109 (12.0%)/264 (29.0%)
ETE (minimal/gross)	375 (41.3%)/46 (5.1%)
Lymphatic invasion	163/831 (19.6%)
Vascular invasion	14/819 (1.7%)
Perineural invasion	17/823 (2.1%)
BRAF mutation	659/777 (84.8%)
Positive LNs	0.6 ± 1.0
Harvested LNs (LNY)	3.3 ± 1.4
Postoperative RAI ablation	547 (60.2%)
T stage	
T1/T2/T3a/T3b	824 (90.6%)/36 (4.0%)/3 (0.3%)/46 (5.1%)
N stage	
N0/N1a	602 (66.2%)/307 (33.8%)
TNM stage	
Stage I/II	808 (88.9%)/101 (11.1%)
Recurrence	46 (5.1%)
Follow-up (months)	127.24 ± 33.6 (range, 5–190)

Data are expressed as number of patients (%), or mean ± SD. Abbreviations: ETE, extrathyroidal extension; LN, lymph node; LNY, lymph node yield; RAI, radioactive iodine; T, tumor; N, node; M, metastasis; and TNM, tumor–node–metastasis.

**Table 2:** Baseline clinicopathologic characteristics according to LNR.

LNR 0.29	Low LNR (*n* = 675)	High LNR (*n* = 234)	*p*-Value
Age (years)	50.21 ± 12.3 (range, 21–83)	47.17 ± 13.2 (range, 20–82)	0.001
Female	551 (81.6%)	182 (77.8%)	0.212
Tumor size (cm)	0.87 ± 0.6 (range, 0.08–5.5)	1.04 ± 0.6 (range, 0.1–3.6)	<0.001
Multifocal (uni/bilateral)	79 (11.7%)/192 (28.4%)	30 (12.8%)/72 (30.8%)	0.131
ETE (minimal/gross)	245 (36.3%)/28 (4.1%)	130 (55.6%)/18 (7.7%)	0.225
Lymphatic invasion	55/559 (9.0%)	108/217 (49.8%)	<0.001
Vascular invasion	10/600 (1.6%)	4/209 (1.9%)	0.762
Perineural invasion	10/603 (1.6%)	7/210 (3.3%)	0.159
BRAF mutation	483/576 (83.9%)	176/201 (87.6%)	0.253
Positive LNs	0.1 ± 0.3	2.0 ± 1.1	<0.001
Harvested LNs (LNY)	3.3 ± 1.4	3.2 ± 1.3	0.485
Postoperative RAI ablation	333 (49.3%)	214 (91.5%)	<0.001
T stage			0.129
T1/T2/T3a/T3b	618 (91.6%)/26 (3.9%)/ 3 (0.4%)/28 (4.1%)	206 (88.0%)/10 (4.3%)/0 (0%)/18 (7.7%)	
N stage			<0.001
N0/N1a	602 (89.2%)/73 (10.8%)	0 (0%)/234 (100%)	
TNM stage			<0.001
I/II	636 (94.2%)/39 (5.8%)	172 (73.5%)/62 (26.5%)	
Recurrence	17 (2.5%)	29 (12.4%)	<0.001

Data are expressed as number of patients (%), or mean ± SD. A statistically significant difference was defined as *p* < 0.05. Abbreviation: LNR, lymph node ratio; ETE, extrathyroidal extension; LN, lymph node; LNY, lymph node yield; RAI, radioactive iodine; T, tumor; N, node; M, metastasis; and TNM, tumor–node–metastasis.

**Table 3:** Univariate and multivariate analyses of the risk factors for recurrence.

	Univariate		Multivariate	
	HR (95% CI)	*p*-Value	HR (95% CI)	*p*-Value
Tumor size	2.026 (1.621–2.533)	<0.001	2.111 (1.596–2.792)	<0.001
ETE				
No	Ref.			
Minimal	2.224 (1.173–4.219)	0.014		
Gross	4.613 (1.789–11.894)	0.002		
Lymphatic invasion				
No	Ref.			
Yes	3.469 (1.861–6.468)	<0.001		
Positive LNs	1.505 (1.251–1.811)	<0.001		
Postoperative RAI ablation				
No	Ref.			
Yes	49.684 (5.076–486.260)	0.001		
LNR	2.832 (1.837–4.365)	<0.001		
<0.29	Ref.		Ref.	
≥0.29	5.173 (2.843–9.414)	<0.001	6.191 (3.176–12.066)	<0.001
T stage				
T1	Ref.			
T2	4.539 (1.904–10.820)	0.001		
T3b	3.417 (1.434–8.142)	0.006		
N stage				
N0	Ref.			
N1a	3.470 (1.907–6.315)	<0.001		
TNM stage				
I	Ref.			
II	2.335 (1.158–4.706)	0.018		

A statistically significant difference was defined as *p* < 0.05. Abbreviation: HR, hazard ratio; CI, confidence interval; LN, lymph node; RAI, radioactive iodine; LNR, lymph node ratio; T, tumor; N, node; and TNM, tumor–node–metastasis. Data are expressed as number of patients (%), or mean ± SD.

**Table 4:** Subgroup analysis for patients with postoperative RAI ablation.

547 Patients with RAI Ablation			
LNR 0.29	Low LNR (*n* = 333)	High LNR (*n* = 214)	*p*-Value
Age (years)	50.26 ± 11.6 (range, 21–80)	47.17 ± 13.2 (range, 20–82)	0.001
Female	279 (83.8%)	167 (78.0%)	0.114
Tumor size (cm)	1.05 ± 0.7 (range, 0.08–5.5)	1.04 ± 0.6 (range, 0.1–3.6)	0.877
Multifocal (uni/bilateral)	35 (10.5%)/111 (33.3%)	27 (12.6%)/69 (32.2%)	0.753
ETE (minimal/gross)	168 (50.5%)/21 (6.3%)	121 (56.5%)/18 (8.4%)	0.142
Lymphatic invasion	48/303 (15.8%)	101/199 (50.8%)	<0.001
Vascular invasion	8/299 (2.7%)	3/191 (1.6%)	0.541
Perineural invasion	8/301 (2.7%)	6/192 (3.1%)	0.786
BRAF mutation	242/285 (84.9%)	165/189 (87.3%)	0.503
Positive LNs	0.2 ± 0.4	2.0 ± 1.1	<0.001
Harvested LNs (LNY)	3.3 ± 1.4	3.2 ± 1.3	0.932
T stage			0.408
T1/T2/T3a/T3b	290 (87.1%)/20 (6.0%)/ 2 (0.6%)/21 (6.3%)	187 (87.4%)/9 (4.2%)/ 0 (0%)/18 (8.4%)	
N stage			<0.001
N0/N1a	273 (82.0%)/60 (18.0%)	0 (0%)/214 (100%)	
TNM stage			< 0.001
I/II	304 (91.3%)/29 (8.7%)	161 (75.2%)/53 (24.8%)	
Recurrence	17 (5.1%)	29 (13.6%)	0.001

Data are expressed as number of patients (%), or mean ± SD. A statistically significant difference was defined as *p* < 0.05. Abbreviation: LNR, lymph node ratio, ETE, extrathyroidal extension; LN, lymph node; T, tumor; N, node; M, metastasis; and TNM, tumor–node–metastasis.

## Data Availability

The data that support the findings of this study are available upon request from the corresponding author. The data are not publicly available due to privacy or ethical restrictions.
